# Genetic Diversity of the Planthopper, *Sogatella furcifera* in the Greater Mekong Subregion Detected by Inter-Simple Sequence Repeats (ISSR) Markers

**DOI:** 10.1673/031.010.5201

**Published:** 2010-06-03

**Authors:** Jia-Ni Liu, Fu-Rong Gui, Zheng-Yue Li

**Affiliations:** Key Laboratory for Agricultural Biodiversity and Pest Management of Ministry of Education, Plant Protection College, Yunnan Agricultural University, Kunming 650201, China

**Keywords:** genetic relationship, ISSR-PCR, migration

## Abstract

The white backed planthopper, *Sogatella furcifera* (Hemiptera: Delphacidae), is a serious pest of rice in Asia. In the present study, inter-simple sequence repeat (ISSR) markers were employed to investigate the genetic diversity and differentiation of 47 populations sampled from 14 prefectures of the Greater Mekong Subregion. A total of 14 selected primers yielded 121 bright and discernible bands, with an average of 8.6 bands per primer. According to the hierarchical analysis of molecular variance (AMOVA), the genetic variation among geographic regions (79.84%) was higher than that of among populations within region (20.16%), and the *F*ST value was 0.72, indicating a high level of genetic differentiation. Neighbor-Joining cluster analysis of the 47 populations showed two major clusters, one consisting of mostly southwestern Yunnan Province and Myanmar populations; and the other one consisting of southeastern and central of Yunnan Province plus Vietnam and Laos populations. No significant positive correlation was observed between genetic and geographic distances by Mantel test (r = 0.2230, p = 0.8448), indicating the role of geographic isolation did not shape the genetic structure of the sampled *S. furcifera* populations. This paper provides useful data for understanding and speculating the migration of *S. furcifera* and reveals available information to develop sustainable strategies for manage this long-range migratory pest.

## Introduction

The white-backed planthopper, *Sogatella furcifera* (Horvath) (Hemiptera: Delphacidae), is widely distributed throughout Asia and is considered a major pest of rice in the region. The nymphs and adults suck the plant sap and reduce plant vigor, delay tillering, stunt, yellow leaves, and shrivel grains (Khan and Saxena 1984), and heavy infestation may cause hopper burn, complete death of the rice plants (Pathak 1968). *S. furcifera* has caused intermittent famines in eastern Asia since ancient times, and became conspicuous in southeast Asia after the so-called Green Revolution of the 1960s (Noda et al. 2008). Because of their long-distance migration, *S. furcifera* can cause sudden devastation to rice. To date, studies on *S. furcifera* have focused mainly upon its biology (Xiao and Tang 2007), occurrence (Seino et al. 1987), varietals resistance (Heinrichs and Rapusas 1985), integrated pest management (Litsinger et al. 2005), and it interactions with *Nilaparvata lugens* (Matsumura and Suauki 2003). However, there is little knowledge of its genetic diversity and population genetic structure.

As *S. furcifera* has been a very serious long-range migratory pest since the early 1970s, many scientists have been engaged in studying its migration with meteorological data. From 1977 to 1980, the main insect populations of China were investigated using high-altitude aerial netting, ship-catching the recapture of the colour-labelled insects, dissection of female ovaries, radar monitoring, atmospheric current analysis etc., and results showed that in the early spring *S. furcifera* came continuously from Indochina Peninsula. *S. furcifera* in the Greater Mekong Subregion, such as Thailand and Vietnam, may carry over by the southwest atmospheric current and its migratory period in south China from March to July. However, small numbers of *S. furcifera* can still be overwinter on spring rice and ratoon rice in southwestern Yunnan Province and southern Guangxi Province (National Coordinated Research Group 1981). Moreover, the rice area in Indochina was the head of Southeast Asia, and the same as in the Greater Mekong Subregion (Bui 1991). Therefore, the pest in China came directly from the Red River Delta, and most of the initial sources were from the Mekong Delta (Wu et al. 1997). This propensity for long-range flight, combined with the small body size of *S. furcifera* and the fact that flight activity is nocturnal, means that it is extremely difficult to observe insect migration while it is in progress (Chapman et al. 2003). However, considering genetic diversity and structure of *S. furcifera* may contribute to speculation about its migratory route and is also essential to the establishment of effective forecasting strategies for this long-range migrate insect.

DNA-based molecular markers have been used in a wide range of taxa. Inter-simple sequence repeat (ISSR) markers, which are cost-effective, rapid and efficiently sensitive, are extremely useful for assessing genetic variability in some species (Gui et al. 2008; Zietkiewicz et al. 1994). In contrast to other dominant markers, such as Random Amplified Polymorphic DNA, the ISSR technique uses longer primers, thus allowing higher annealing temperature and greater reproducibility of the DNA fragments (Fang et al. 1997). The high degree of polymorphism, low cost, and good repeatability of ISSRs have allowed the successful detection of intra-specific polymorphisms and characterization of genetic diversity in various species, such as peanut, *Arachis hypogaea* (Raina et al. 2001), two species of cyclically parthenogenetic aphids, *Acyrthosiphon pisum* and *Pemphigus obesinymphae* (Abbot et al. 2001), and the mosquito *Aedes aegypti* (Abbot et al. 2001).

The fact that the population of *S. furcifera* in China has not been suppressed may be due to the ineffective coordination of implementing the control strategy, as well as the lack of comprehensive knowledge (including population structure and diversity) of the pest. A sound understanding of the genetic diversity, migration/dispersal patterns, and the environmental adaptability of *S. furcifera* is essential to the development of rational control strategies. The present study was designed to use ISSR analysis for investigating the genetic structure as well as diversity of *S. furcifera* among the geographic regions in the partial Greater Mekong Subregion. The main objectives of the study were to (i) assess bio-geographic relationships and genetic similarities across several populations in Yunnan Province and its adjacent southeastern Asian countries; and (ii) provide useful information in modeling and forecasting outbreaks of *S. furcifera* and in designing sustainable strategies to manage the pest.

**Figure 1.**
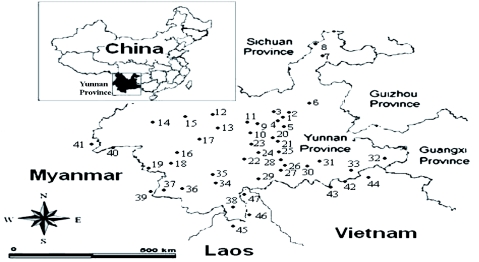
Locations of the 47 *Sogatella furcifera* populations in the partial Greater Makong Subregion. Numbers represent the different populations. 1–5: Kunming (KM); 6: Qujing (GJ); 7–8: Zhaotong (ZT); 9–11: Chuxiong (CX); 12–13: Dali (DL); 14–15: Baoshan (BS); 16–19: Lincang (LC); 20–24: Yuxi (YX); 25–30: Honghe (HH); 31–33: Wenshan (WS); 34–37: Puer (PE); 38: Xishuangbanna (XSBN); 39–41: Myanmar (MYA); 42–44: Vietnam (VIE); 45–47: Laos (LAO)High quality figures are available online.

## Materials and Methods

### Sampling

A total of 47 populations of *S. furcifera* were sampled across 11 geographical regions in Yunnan Province and three Southeast Asian countries, including Vietnam, Laos, and Myanmar. The longitude, latitude, and altitude of each sampling population, sample size, and collection dates were recorded (Figure 1; Appendix — available online). Approximately 30 individuals per population were collected and the collected samples were stored in 80% alcohol until ISSR analysis.

### DNA extraction

Total genomic DNA was isolated from *S. furcifera* using a modified SDS method (Wang et al. 2001). The DNA concentration and 260/280 nm absorbance ratio were determined using a GeneQuant RNA/DNA calculator spectrophotometer (Pharmacia Biotech, www.apbiotech.com). All samples were stored at -20°C until needed.

### ISSR-PCR amplification

Sixty-seven ISSR primers were selected from the ISSR primer set (UBC primer set #9) developed by the University of British Columbia Biotechnology Laboratory (www.biotech.ubc.ca) and synthesized by Sangon Biological Engineering Technique & Service, Co. Ltd. (www.sangon.com). These primers were initially screened, and the 14 primers that produced bright, clear, and reproducible fragments were utilized for further study (Table 1).

Each PCR amplification reaction mixture consisted of 2 µl reaction buffer, 2.5 mM/L Mg^2+^, 2 µM/L dNTPs, 0.8 *µ MIL* primer, 1 U *Taq* DNA polymerase (TaKaRa, www.takarabio.com), and 30 ng DNA templates in a total volume of 20 µl, and 2.0% of deionized formamide was added to the PCR mixture to increase band clarity. Amplification was performed in a Mastercycler Gradient (Eppendorf, www.eppendorf.com) under the following cycle profile: 4 min at 94°C, followed by 35 cycles of 1 min at 94°C, 1 min annealing (temperature depending on primers used) (Table 1), and 2 min extension at 72°C, ending with 10 min at 72°C for a final extension. The PCR products were separated on 2% agarose gels in 0.5 × TBE buffer and detected by staining with GeneFinder. Band size was compared with a 100 bp DNA ladder (TaKaRa), and determined by spectro-photometry using an ImageQuant 300 (Beckman Instruments Inc., www.beckmancoulter.com)

### Data analysis

The ISSR bands were analyzed to estimate the genetic variations among and within populations studied. The banding patterns were recorded using a gel documentation system (Bio-Rad Gel Doc 1000, www.biorad.com). Amplified fragments were scored for the presence or absence of bands (1 = present; 0 = absent; 9 = not amplified, missing value). Since ISSR markers were dominantly inherited, each band was assumed to represent the phenotype at a single biallelic locus (Williams et al. 1990). Bands with differing intensity were treated equally, but only bright and discernible fragments ranging from 220 to 2000 bp were included in the statistical analysis.

To evaluate the discriminatory power of molecular markers, polymorphic information content and marker index were calculated according to Gui et al. (2008). The ISSR molecular data were elaborated using the NTSYS-pc (Numerical Taxonomy System) version 2.10 computer program (Rohlf 2002). The SEVIQUAL (similarity for qualitative data) program was used to calculate the genetic similarities. Similarity matrices were then converted into distance matrices (distance = 1 - similarity). Based on these matrices, dendrograms were constructed using the Neighbor-Joining (NJ) method. In addition to NJ cluster analysis, UPGMA (unweighted pair-group method with arithmetic averages) was performed on the same data sets. All the computations were performed using NTSYS-pc software.

The bootstrap analysis was performed with 500 replicates in NJ trees using the FreeTree software (available at: http://www.natur.cuni.cz/∼flegr/freetree.htm). In order to estimate the congruency among dendrograms, cophenetic values (rcp) based on the results of the NJ cluster and UPGMA cluster analysis were calculated to measure the quality of the clustering (Rohlf and Sokal 1981). The cophenetic matrices for each index type were computed and compared using the Mantel matrix correspondence test. This test yielded a product moment correlation (r) that provided a measurement of relatedness between two matrices.

In order to partition the total phenotypic variance into within and among populations, the non-parametric Analysis of Molecular Variance (AMOVA) program 1.5 was also applied as described by Excoffier (1993), where the variation component was partitioned among individuals within population, among populations within region, and among regions. Then a permutational procedure (i.e. 1000 random permutations) was used to provide tests of significance for each of the hierarchical variance components based on the original inter-individual squared-distance matrix. Homogeneity of molecular variance among populations was tested with Bartlett's statistics. The input files for AMOVA were prepared with the aid of AMOVA-PREP version 1.01(Miller 1998).

**Table 1.**
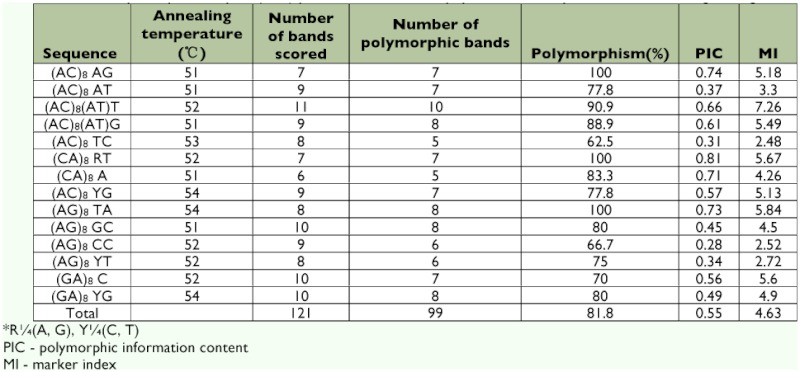
Inter-simple sequence repeat (ISSR) primers for S. furcifera populations in the partial Greater Makong Subregion.

**Table 2:**
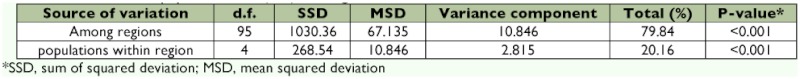
AMOVA in 14 populations of *S.* *furcifera* using 121 ISSR markers

Geographical distances of pairs of populations were calculated using the latitude, longitude, and elevation of each population. The Mantel Z-statistic (1000 permutations; routine MXCOMP in NTSYS) was used to test the correlation between geographical distances and genetic distances (Mantel 1967). As one of the most important methods of ordination analysis, principal coordinate analysis (PCOA) was performed using the NTSYS-pc version 2.10 software (Rohlf 2002) to examine the resolving power of the ordination. It constructed a new set of orthogonal coordinate axes maximum variance in as few dimensions as possible.

## Results

### SR profile

**Figure 2.**
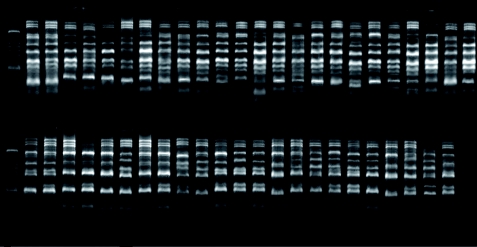
ISSR PCR amplification patterns of *Sogatella furcifera* populations using primer (AC)8(AT)T. (M: 100 bp DNA ladder; lane 1–47 same as the site number of Appendix - available online in supplementary material)High quality figures are available online.

Fourteen ISSR primers were selected from a total of 67 on the basis of clarity, usability, and reproducibility of their banding patterns; the data are shown in Table 1. The 14 primers produced a total of 121 bright and discernible bands, 81.8% (99 bands) of which were polymorphic. The number of bands produced by individual primers was in the range of 6–11 with an average of 8.6. The size of the polymorphic bands ranged from 220 bp to 2000 bp. The representative banding patterns are shown in Figure 2. The primers differed greatly in their potential usability as indicated by the number of securable amplified bands, e.g. the primer (AC)8(AT)T produced as many as 11 bands, while primer (CA)_8_ A amplified only 6 bands. The average polymorphic information content varied from 0.28 (AG)_8_CC) to 0.74 (AC)_8_AG), whereas the marker index ranged from 2.48 (AC)_8_TC) to 5.84 (AG)_8_TA). The mean polymorphic information content and marker index of the 14 primers was 0.55 and 4.63, respectively. The ISSRs that exhibited a high polymorphic information content value, together with a higher multiplex ratio, were likely to be efficient for the analysis of intra-specific genetic variation in a species like *S. furcifera* for which no prior sequence information was available.

### Genetic diversity of *S. furcifera*

To assess the overall distribution of genetic diversity, the AMOVA program was used to analyze the distance matrix, the data are shown in Table 2. AMOVA provides *F*ST of population differentiation, which is equivalent to an *F*ST statistics when the degree of relatedness among the genetic variants is evaluated (Belaj et al. 2004). AMOVA analysis showed highly significant (p < 0.001) genetic differentiation among regions. A large proportion of genetic variation (79.84%) resided among regions, whereas only 20.16% resided among populations within regions. The *F*ST value showed a much higher differentiation (*F*ST =0.72) among regions, indicating a high level of genetic differentiation.

### Cluster and coordination analysis

The similarity matrices calculated from the polymorphic ISSR bands showed highly variable genetic distances among the different populations. The genetic distance was the highest (1.1052) between the northeast population (Daguan, Zhaotong) and Red River Delta population (Yuanyang, Honghe) in Yunnan Province, and the lowest (0.1475) between southern population (Mengzi, Honghe) in Yunnan Province and Maten population in Vietnam.

**Figure 3.**
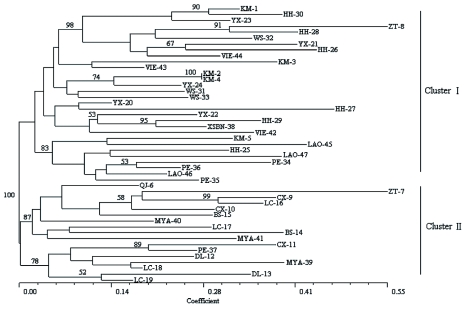
Neighbour-joining cluster analysis of the 47 populations of *Sogatella furcifera* generated from the Dice distance matrix. Only bootstrap values over 50% are shown. The numbers refer to Appendix — available online in supplementary material. High quality figures are available online.

The dendrogram generated from the NJ cluster analysis showed the genetic relationship among 47 *S. furcifera* populations; the data are shown in Figure 3. The populations collected from similar geographic regions generally grouped in the same cluster or nearby clusters. Two major clusters, one consisting of mostly southwestern populations of Yunnan Province and Myanmar populations; and the other one consisting of southeastern and central populations of Yunnan Province (including all the Red River Delta populations), plus Vietnam and Laos populations, were visible in NJ cluster analysis. The UPGMA tree showed a similar pattern of clustering with the NJ tree, and the results of the Mantel test indicated a highly significant cophenetic correlation (r = 0.7748, p = 0.0001) between the NJ tree and the UPGMA tree.

**Figure 4.**
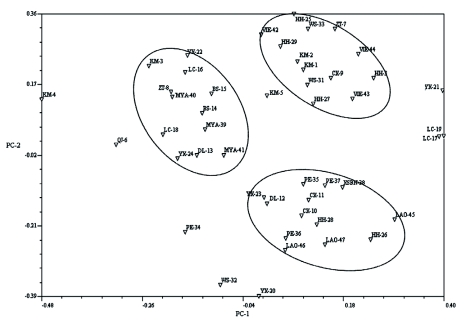
Association among the 47 populations of *Sogatella furcifera* revealed by PCOA analysis performed on Dice genetic distance index calculated from ISSR data. The site numbers refer to Appendix — available online in supplementary material. High quality figures are available online.

The results of PCOA showed three main groups in the two dimensional PCOA; the data are show in Figure 4. Group 1 included populations from Myanmar and adjacent Lingcang and Baoshan prefectures of Yunnan Province (Cluster II in the NJ tree). Group 2 included populations from Laos, small partial populations of Red River Delta and Puer prefecture of Yunnan Province (the bottom populations of Cluster I in NJ tree, see Figure 3), and Group 3 included populations from northern Vietnam, most of the Red River Delta and Wenshan prefecture of Yunnan Province (the upper populations of Cluster I in NJ tree, see Figure 3). The first two components of PCOA explained 15.94% of the total variation, and the first three components explained 22.62% of the total variation (data not shown). The results of PCOA and NJ tree tend to be uniform in whole, reflecting the geographical distribution of *S. furcifera* in the Greater Mekong Subregion. However, there were a few populations that did not fit into any group (e.g. site WS32 and YX20 did not fall into any group in PCOA), and the Red River Delta populations were split into Group 2 and Group 3 in the PCOA while belong to cluster I in NJ tree. The lack of fit was also reflected in the results of the Mantel test, where the geographical and genetic matrices did not have an overall correlation (r = 0.2230, p = 0.8448), indicating the role of geographic isolation did not shape the present population genetic structure of *S. furcifera.*

## Discussion

The ISSR marker approach provided means of examining bio-geographic relationships and genetic similarity within and among populations of *S. furcifera* in Yunnan Province, China and the potential original areas of this migratory pest in the adjoining south and southwest countries. ISSRs can be informative at various levels of genetic variation (Hess et al. 2000), and it can be advantageous when time and material costs preclude the development of more robust markers (e.g., locus-specific SSRs). ISSR markers are also highly reproducible due to stringent annealing temperatures, long primers, and low primer-template mismatch (Wolfe et al. 1998). While the detection using more sensitive techniques (autoradiography or silver staining) on Polyacrylamide gels may increase the resolution of co-migrating fragments (Godwin et al. 1997) in comparison with allozymes and Random Amplified Polymorphic DNA, ISSRs can also reveal polymorphisms without more elaborate detection protocols (Esselman et al. 1999). Thus, for biological questions where genomic fingerprinting is appropriate (Abbot et al. 2001), ISSR is a valuable marker for rapid, large-scale screening of genetic variation in animal populations. The 14 selected ISSR primers were di-nucleotides and mostly poly (AC) or poly (AG). This was in correspondence with studies of other animal species (such as *Drosophila melanogaster*) where the poly (AC) and poly (AG) repeats are common repeat motifs across animal groups (Schug et al. 1998).

Between the NJ and UPGMA clustering analysis, the dendrogram derived from the NJ method was preferred because it minimized the sum of branch lengths at each stage of clustering the operational taxonomic units and started with a star-like tree, which was less affected by the presence of admixture among populations (Ruiz-Linares 1994). The PCOA analysis proved relatively regional distribution of *S. furcifera.* However, a few populations were not grouped within its adjacent populations, which suggested that there was genetic heterogeneity within each geographical region, and further suggested that most of the *S. furcifera* in Yunnan are migrated from the adjoining south and southwest countries via different routes or at different times, and a small part of them may origin from the offspring of the overwintering individuals in southern Yunnan Province. Because *S. furcifera* is a migratory insect pest, the seasons when these insects were collected are very important. In the present study, all of the samples were collected from May to July. As *S. furcifera* in the Greater Mekong Subregion may carry over by the southwest atmospheric current and its migratory period in south China is from March to July (National Coordinated Research Group for Whiteback planthopper 1981). So most of the pests collected may be migratory individuals.

Based on AMOVA analysis, 79.84% of the total variation was found among regions, while 20.16% variance was attributable to population divergence within the regions. The population genetic structure of a species is affected by a number of evolutionary factors including its mating system, gene flow, and its mode of reproduction as well as its natural selection (Hamrick and Godt 1989), and the mating system plays a critical role for the population genetic structure. ISSR markers can potentially distinguish many individuals, but it cannot provide direct information on the mating system due to their dominant nature of inheritance (Wolfe and Liston, 1998). In addition, for the highly migratory insect, one has have to consider their population characteristics such as insecticide resistance, virulence against resistant rice varieties, and winged response to density which could vary considerably among populations collected from different geographic locations.

Migration is a fundamental population process and a common feature of insect life cycles, the study of which is crucial to understanding the dynamics and persistence of populations of insects (Dingle 1996). The first study of migration ecology and distribution of the individual species in the complex was conducted by Chapman et al. (2006). They studied seasonal variation in the migration strategies of the green lacewing *Chrysoperla cornea* species complex, and demonstrated the migratory capabilities of the individual species comprising the *C. cornea* group of lacewings, and indicated that understanding the population ecology of an insect species is necessary to investigate the complete migration syndrome. Xian et al. (2007) used significant El Nino-Southern Oscillation indices as key factors to build forecasting models for the early immigration of the brown planthopper, *Nilaparvata lugens* by step-wise multiple linear regression analysis. The results showed that these indices can implicate the medium and long-term forecast of *N. lugens* population dynamic.

For the migratory insect, genetic variation is found in all components of the migratory syndrome, and selection for migration results in a change in the frequency of expression of these components, which can be analyzed and predicted using the mathematics of quantitative genetics (Roff and Fairbairn 2007). Variability and the genetic basis for migratory behaviors in a spring population of the aphid, *Aphis gossypii* in the Yangtze River Valley of China was investigated by Liu et al. (2008). The tethered flight capacity, takeoff frequency, and takeoff angle of winged *A. gossypii* were measured, and the genetic basis of population differentiation in migration was investigated through bi-directional selection and cross-breeding experiments. The study provided further evidence that the intra-population variability of migratory behaviors in *A. gossypii* is of genetic origin, and that the migratory line produces winged offspring more readily than the sedentary line. Llewellyn et al. (2003) used microsatellites to study migration and genetic structure of the grain aphid, *Sitobion avenae,* in Britain related to climate and clonal fluctuation. The data sets support the view that the insect is highly migratory and an accurate picture relating genetic variability to flight behavior, including migratory ambit, in this group of insects can be built up using microsatellite markers.

The present study is the first attempt of assessing the genetic diversity of *S. furcifera* using the ISSR marker technique. It demonstrated the validity and suitability of using ISSR markers to detect the genetic variation among populations of *S. furcifera* from different regions. Although there are difficulties in using conventional approaches to discern the accurate migratory route of *S. furcifera* (e.g. fluorescent marker dyes, radio-isotopes) not only due to the special geographic environment and meteorologic condition of Yunnan Province (high plateau, high altitude, complex hypsography, and various climate conditions), but also as their small size, short lifespan, large population sizes, rapid aerial population dilution and the very long distances over which these insects may fly (Loxdale et al. 1993), the application of ISSR markers has the potential to overcome many of these challenges and provides an overall understanding of population relationships of the species. From a fundamental point of view, since genetic structuring of populations reflects the interaction of genetic drift, mutation, migration and selection, *S. furcifera* are of particular interest in this regard. In addition to demonstrating the usefulness of ISSR markers for DNA profiling, the genetic structures among populations of *S. furcifera* analyzed in this study enable us to infer its evolutionary relationship. Based on the results of the study, it can be speculated that *S. furcifera* migrates to Yunnan Province primarily by two routes, one from northern Vietnam and Laos to the Red River Delta which is in the southeastern Yunnan Province, China; and the second one from Myanmar to the southwestern areas of Yunnan Province such as Lincang and Baoshan prefectures. After that, these two migrators disperse, spreading and finally causing outbreaks in the whole rice area of the Province, even to the whole country. This speculation was supported by Wu et al. (1997), as they indicated that *S. furcifera* migrated to China via two routes: one was to southwestern Yunnan by southwest monsoon from the northern Thailand and Myanmar; and the other one was to the Red River Delta in Yunnan and Guangxi, Guangdong Province due to the southwest monsoon from Indochina. The present data does not clearly indicate the migration pattern of *S. furcifera* as there still is a dearth of information on genetic architecture. Phenotypic variation in migratory propensity has long been known, but the genetic basis of such variation is still relatively unexplored. Even more important, although it is recognized that migration is not a single trait but a suite of traits that include both larval and adult components, more data are needed on the functional and genetic relationships among traits (Roff and Fairbairn 2007). Therefore, further studies in wing dimorphisms of the insect may lead to more effective research into migratory behavior and population dynamics in various geographic regions, furthering speculation on possible diffused direction and the timing of sudden break out. Thus, information on the migration patterns of important agriculture insect is essential to developing sustainable pest management strategies.

## Abbreviations

ISSR: inter-simple sequence repeats;
NJ: Neighbor-Joining;
NTSYS: Numerical Taxonomy System;
PCOA: principal coordinate analysis;
UPGMA: unweighted pair-group method with arithmetic averages
